# Psychological disorder detection: A multimodal approach using a transformer-based hybrid model

**DOI:** 10.1016/j.mex.2024.102976

**Published:** 2024-09-24

**Authors:** Debadrita Ghosh, Hema Karande, Shilpa Gite, Biswajeet Pradhan

**Affiliations:** a Symbiosis Centre of Applied AI (SCAAI), Symbiosis International (Deemed) University, Pune 412115, India; b Artificial Intelligence & Machine Learning Department, Symbiosis Institute of Technology, Symbiosis International (Deemed) University, Pune 412115, India; c Centre for Advanced Modelling and Geospatial Information Systems (CAMGIS), School of Civil and Environmental Engineering, Faculty of Engineering and Information Technology, University of Technology Sydney, Australia

**Keywords:** Psychological distress, Depression, Mental disorder, Deep learning, AI, Hybrid fusion technique integrating Deep Learning, NLP and Machine Learning algorithm

## Abstract

Detecting psychological disorders, particularly depression, is a complex and critical task within the realm of mental health assessment. This research explores a novel approach to improve the identification of psychological distresses, such as depression, by addressing the subjectivity, complexity, and biasness inherent in traditional diagnostic techniques. Using multimodal data, such as voice characteristics and linguistic content from participant interviews, we developed a Transformer-Based Hybrid Model that combines advanced natural language processing and deep learning approaches. This model provides a complete assessment of an individual's psychological well-being by merging aural cues and textual data. This study investigates the theoretical underpinnings, technical complexities, and practical applications of this model in the context of psychological disorder detection. Additionally, the model's design and implementation details are thoroughly documented to ensure replicability by other researchers.

• A unique way of strengthening emotional ailments (focusing on depression).
• Transformer-Based Hybrid Model is proposed using multimodal data from interviews of participants.
• The model integrates voice characteristics (aural cues) and linguistic content (textual data).
• Comparative analysis of this research with existing approaches.

## Graphical abstract

**Figure:**
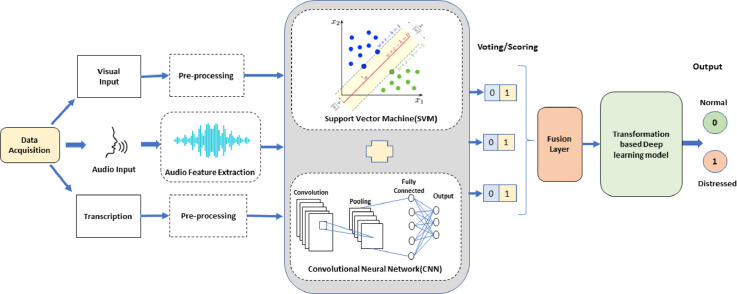

Specifications table

**Table:** 

Subject area:	Computer science
More specific subject area:	Application of Artificial Intelligence in Healthcare Domain
Name of your method:	Hybrid fusion technique integrating Deep Learning, NLP and Machine Learning algorithm
Name and reference of the original method:	Hybrid CNN-SVM classifier. It describes employing a hybrid CNN-SVM model on audio input using same dataset. This concept was taken as reference and our model was built with SVM-CNN fusion for all three modalities. The results of the existing and our model was also compared. Reference: Saidi, Afef & Ben Othman, Slim & Ben Saoud, Slim. (2020). Hybrid CNN-SVM classifier for efficient depression detection system. 10.1109/IC_ASET49463.2020.9318302.
Resource availability:	Data set was accessible from the following source: https://dcapswoz.ict.usc.edu/).

## Background

Mental disorders are becoming increasingly prevalent, posing a major source of distress in people's lives and impacting society's well-being. Psychological disorders are multifaceted illness influenced by individual risk factors, lifestyle, and a range of economic, social, and behavioral relationships [[1], [8], [9], [15]]. Psychological health can be understood as an individual's social, emotional, and spiritual well-being [[2], [10], [17]]. It can be affected by a variety of mental stress factors or psychological conditions that negatively impact a person's cognitive function, emotions, and social connections. To address these illnesses, proper and timely assessment is required to distinguish one from another [[2], [16], [18]].

The precise detection and diagnosis of psychological problems are critical in the field of mental health. Millions of people worldwide suffer from psychological problems, with serious implications for their quality of life and overall health. Timely and accurate identification of these illnesses not only allows for early intervention but also plays a critical role in developing effective treatment techniques.

Technological advances, such as smart phones, web media, digital wearables, and neuroimaging, have enabled health practitioners, researchers and doctors to acquire a large ensemble of data at an increasing pace [[3], [12], [19], [21]]. Illness, like depression, dementia, and anxiety negatively affect health and thus necessitate more effective identification techniques to allow for the earliest treatment [[4], [20]].

Novel approaches have been developed through the confluence of psychology and artificial intelligence, recognizing the vital need for enhanced tools in this sector. Computational algorithms use several input modalities for the automatic detection of sadness, such as user-generated text on social media [[5], [13], [17]], interview transcripts [[6], [10]], and the interviewee's voice signal. Some of the most extensively studied applications of artificial intelligence in mental health treatment are currently detection systems [[7], [22], [23]].

## Method details

This section provides a comprehensive overview of the methodology employed to detect psychological distress using multimodal data. The methodology integrates data collection, preprocessing, feature extraction, and model development, focusing on text, audio, and visual modalities.

## Data description

The dataset used in this study is accessible at https://dcapswoz.ict.usc.edu/, but requires consent and licensing due to ethical reasons. It includes recordings from clinical interviews and assessments involving participants diagnosed with various mental health disorders and a control group without diagnosed disorders. The dataset comprises multiple modalities, including text (transcriptions), audio, and video recordings.

## Data preprocessing

The preprocessing phase involved multiple steps tailored to each modality:

• **Audio**: The audio recordings underwent noise reduction, segmentation, and transcription. These transcriptions were then used to derive text-based features. The dataset was initially imbalanced, with a 7:3 ratio between non-depressed and depressed classes. To address this, we applied up sampling techniques to balance the dataset.
• **Text**: Text-based features were extracted from the voice transcriptions using Natural Language Processing (NLP) techniques. Tokenization and segmentation were performed at the word and sentence levels. Significant words were identified using TF-IDF representation, and various linguistic features such as word counts, sentence length, and vocabulary diversification were analyzed.
• **Video**: The video modality involved extracting features related to facial gestures, gaze, and poses. Specifically, 68 2D and 3D facial points, 24 facial movement AUs, 16 gaze-related features, and 10 pose features were captured, resulting in a total of 388 video features.

## Feature extraction

We extracted features from the three data types: auditory, visual, and text-based. Time-stamps were retained to preserve time-dependent interactions, enabling contextual learning. Sentence-level integration was necessary to align audio, textual, and visual features, as each modality uniquely influences the final outcome.

## Model development

A Transformer-based deep learning (DL) hybrid model was developed to detect psychological distress. The model architecture is divided into two stages:

• **Text Pipeline**: Transcriptions were fed into a text pipeline that processed the data through a transformer-based DL system for sentence categorization.
• **Speech and Visual Pipelines**: These pipelines extracted information from the patient's voice and facial gestures, which were then classified using statistical methods. Outputs from each modality were fused using a trained classifier, which produced the final predictions.

**Gating Mechanism**: Post feature extraction, a gating mechanism was applied to regulate the influence of each modality on the final output. Weight vectors were assigned to each modality to control and learn input translation to subsequent layers. Audio/visual vectors were filtered before concatenation to ensure relevant features were extracted (Table 1).

**Table 1:** Sequential breakdown of model architecture.

Layer (type)	Output Shape	Param
Video_input (InputLayer)	(None, 1700, 388)	0
Audio_input (InputLayer)	(None, 1700, 74)	0
highway_9 (Highway)	(None, 1700, 388)	1,620,288
highway_6 (Highway)	(None, 1700, 74)	262,552
highway_10 (Highway)	(None, 1700, 388)	1,620,288
highway_7 (Highway)	(None, 1700, 74)	262,552
highway_11 (Highway)	(None, 1700, 388)	1,620,288
Text_input (InputLayer)	(None, 1700, 301)	0
highway_8 (Highway)	(None, 1700, 74)	262,552
dense_7 (Dense)	(None, 1700, 200)	77,800
dense_9 (Dense)	(None, 1700, 150)	45,300
dense_6 (Dense)	(None, 1700, 74)	5550
dense_8 (Dense)	(None, 1700, 74)	14,874
dense_10 (Dense)	(None, 1700, 74)	11,174
concatenate_1 (Concatenate)	(None, 5100, 74)	0
lstm_1(LSTM)	(None, 128)	103,936
dense_11(Dense)	(None, 1)	129
Video_input (InputLayer)	(None, 1700, 388)	0
Audio_input (InputLayer)	(None, 1700, 74)	0
highway_9 (Highway)	(None, 1700, 388)	1,620,288
Total parameters-5907,283		

## Model description

The model is designed for sequence classification tasks, with input sequences comprising 74 features and a length of 6000. The architecture was implemented using the Keras Sequential API. The model was compiled with the Adam optimizer, accuracy as the performance metric, and binary cross-entropy as the loss function.

**Hybrid-Fusion Technique**: A unique hybrid strategy was employed, integrating algorithmic fusion techniques. Initially, basic machine learning classifiers were applied to each modality individually. A second-stage SVM model was trained on the scores or labels obtained from the initial models to perform late fusion. Features integrated with time-stamps were processed through sentence and word-level LSTM and BiLSTM models. The final predictions were made by concatenating scores from both new and original modalities.

**Model Variations**:

• **SVM, Random Forest, and CNN**: Individual SVM and Random Forest classifiers were applied to each modality. A CNN model with six layers was developed, with specific layers for each modality type (Conv2D for text, Conv1D for audiovisua). The final layers used ReLU and sigmoid activation functions.
• **LSTM and BiLSTM**: Gating of auditory and visual features was performed using Highway layers. LSTM and BiLSTM models were employed, with sentence and word-level gating for aligned data. Dimensionality reduction was conducted using dense layers, followed by BiLSTM for final output generation.
• **BERT**: A pretrained BERT model was fine-tuned for binary classification tasks. This model processed transcriptions through a BERT-deep learning hybrid architecture, with final scores ensembled with other models' outputs.

## Dataset specifics

The DAIC-WOZ dataset, used in this study, includes multimodal recordings of clinical interviews and assessments. The dataset is characterized by:

• **Text Modality**: Transcribed data in CSV format, including timestamped patient conversations, with speaker classifications and emotional expressions annotated.
• **Audio Modality**: Audio features recorded at 10 ms intervals with a sampling rate of 100 Hertz, including twelve MFCCs and other auditory cues.
• **Video Modality**: Facial gestures, gaze, and pose features extracted from video recordings, resulting in 388 total video features.

The dataset size is approximately 156 GB, consisting of recordings from 189 interview sessions (59 depressed, 130 non-depressed), ranging from 7 to 33 min each (Table 2).

**Table 2:** Results of the work done by our model using DAIC-WoZ dataset.

Model	Modality	Precision	Recall	F1 - Score
Late Fusion using SVM	Text + Audio + Video	0.45	0.39	0.42
CNN	Audio	0.35	0.84	0.50
	Text	0.57	0.62	0.59
	Video	0.38	0.58	0.35
LSTM	Audio	0.58	0.46	0.47
	Text	0.66	0.68	0.67
	Video	0.49	0.68	0.57
LSTM without Gating at Sentence-Level	Audio +Text	0.72	0.36	0.48
	Text + Video	0.61	0.47	0.48
	Audio + Text + Video	0.63	0.49	0.50
LSTM with Gating at Sentence-Level	Text + Video	0.64	0.54	0.56
	Text + Audio	0.64	0.66	0.65
	Audio + Text + Video	0.64	0.64	0.65
LSTM with Gating at Word-Level	Audio + Text + Video	0.49	0.66	0.56
BILSTM (Word-Level)	Text + Audio + Video	0.79	0.321	0.19
BERT	Text + Audio + Video	0.84	0.73	0.78

## Method validation

The dataset was initially imbalanced, with a 7:3 ratio favoring the non-depressed class. To ensure robustness, we applied up sampling to balance the dataset. The models were evaluated using precision, recall, and F1-score, providing a comprehensive assessment of their performance.

**Hybrid SVM-CNN Model**: Our proposed hybrid SVM-CNN model achieved a precision of 0.34, a recall of 0.83, and an F1-score of 0.49 for the audio modality. For the text modality, it achieved a precision of 0.56, recall of 0.61, and F1-score of 0.58. The video modality yielded slightly lower scores with a precision of 0.38, recall of 0.58, and F1-score of 0.35. The combined multimodal approach improved the model's accuracy, achieving a performance of 51.54 %.

**LSTM and BiLSTM Models**: LSTM models incorporating gating mechanisms at different levels demonstrated improved performance. The LSTM with sentence-level gating achieved a precision of 0.64, recall of 0.66, and F1-score of 0.65 when combining audio, text, and video modalities. The BiLSTM model exhibited a strong bias towards the depressed class but performed well overall, particularly when handling fused modalities.

**BERT Model**: The BERT-based model excelled in handling fused modalities, outperforming other models with a precision of 0.84, recall of 0.73, and F1-score of 0.78. This model's superior performance highlights the effectiveness of transformer architectures in capturing the nuances of multimodal data (Table 3).

**Table 3:** Comparisons of results from different existing works using DAIC WoZ data.

Name	Precision	Recall	F1-Score	Accuracy (%)
Gaussian-Staircase Model [11]	0.570	**–**	**–**	**–**
DepAudioNet [14]	0.520	1.00	0.350	**–**
Random Forest Algorithm [24]	0.57	0.50	0.57	61
Hybrid CNN-SVM classifier [25]	0.550	0.860	0.52	58
SVM-CNN (Audio)	0.34	0.83	0.49	51.54
SVM-CNN (Text)	0.56	0.61	0.58	49.8
SVM-CNN (Video)	0.38	0.58	0.35	41.7
BiLSTM(Our Final Model1)	0.70	0.97	0.81	68
BERT(Our Final model2)	0.84	0.73	0.78	81.6

## Comparative analysis

Our method was compared against several existing models, including DepAudioNet, Random Forest, and other hybrid classifiers. The Hybrid CNN-SVM model from previous studies, which utilized similar datasets, achieved an accuracy of 58 %, while our approach with BiLSTM reached 68 %, and BERT-based models attained 81.6 %. DepAudioNet, known for its high recall, suffered in precision and F1-score, indicating potential issues with false positives. In contrast, our model exhibited a more balanced performance, effectively minimizing false positives while maintaining high recall.

## Limitations

**Data Size and Multimodal Integration**: The large data size poses challenges in terms of storage, processing, and analysis. Handling such extensive data requires significant computational resources, which may not be readily available to all researchers. Moreover, despite the hybrid model's strength in integrating text, audio, and visual data, the complexity of aligning these modalities effectively may introduce noise.

**Overfitting**: While efforts were made to prevent overfitting, including the use of techniques like dropout and early stopping, the model's complexity and the limited size of the training data could still lead to overfitting. This issue highlights the importance of extensive cross-validation and the potential need for more robust datasets in future studies.

**Subject Sensitivity**: The model's ability to accurately interpret emotional cues and context may be limited by the quality and diversity of the input data. Emotional expressions in speech and facial gestures vary significantly across individuals, potentially affecting the model's predictions.

## Ethics statements

The dataset used in this study is accessible at https://dcapswoz.ict.usc.edu/, but requires consent and licensing due to ethical reasons. The authors have acquired necessary permissions from the dataset owners and its proof is also attached in the submission documents.

## CRediT authorship contribution statement

**Debadrita Ghosh:** Conceptualization, Methodology, Investigation, Writing – original draft. **Hema Karande:** Writing – review & editing, Validation, Supervision. **Shilpa Gite:** Writing – review & editing, Validation, Supervision. **Biswajeet Pradhan:** Visualization, Writing – review & editing, Funding acquisition.

## Declaration of competing interest

The authors declare that they have no known competing financial interests or personal relationships that could have appeared to influence the work reported in this paper.

## Data Availability

Data will be made available on request.
